# An analysis of two indigenous reproductive health illnesses in a Nahua community in Veracruz, Mexico

**DOI:** 10.1186/1746-4269-8-33

**Published:** 2012-08-22

**Authors:** Vania Smith-Oka

**Affiliations:** 1 Anthropology Department, 611 Flanner Hall, Notre Dame, IN, 46556, USA

**Keywords:** Indigenous ethnomedicine, Women’s health, Reproductive health, Medicinal plants, Mexico, Nahua

## Abstract

**Background:**

This article describes the local concepts indigenous Nahua women hold regarding their reproduction. Specifically it provides a description of two indigenous illnesses—*isihuayo* and *necaxantle*, it discusses their etiology, symptoms, and treatments, and it analyzes them within the local ethnomedical framework and sociopolitical context. A perception of female vulnerability is shown to be an underlying shaper of women’s experiences of these illnesses.

**Methods:**

This research took place in a small Nahua village in Mexico. Qualitative data on local perceptions of these illnesses were collected by a combination of participant observation and interviews. Ethnobotanical data was obtained through interviews, and medicinal plants were collected in home gardens, fields, stream banks, and forested areas. The total study population consisted of traditional birth attendants (N = 5), clinicians (N = 8), and laywomen (N = 48).

**Results:**

Results showed that 20% of the village women had suffered from one or both of these illnesses. The article includes a detailed description of the etiology, symptoms, and treatments of these illnesses. Data shows that they were caused by mechanical, physical, and social factors related to a woman’s weakness and/or lack of support. Traditional birth attendants often treated women’s illnesses. Five medicinal plants were salient in the treatment of these illnesses: *Ocimum basilicum* L., *Mentzelia aspera* L., *Pedilanthus tithymaloides* (L.) Poit., and *Piper umbellatum* L. were used for *isihuayo*, while *Solanum wendlandii* Hook f. was used for *necaxantle*.

**Conclusions:**

The research on these two ethnomedical conditions is a useful case study to understanding how indigenous women experience reproductive health. Reproductive health is not simply about clinically-based medicine but is also about how biomedicine intersects with the local bodily concepts. By describing and analyzing indigenous women’s ill health, one can focus upon the combination of causes—which extend beyond the physical body and into the larger structure that the women exist in.

## Background

The traditional medicine of indigenous groups of Mexico has been very well studied over the past decades [1-7]. Many of these studies show the important role that medicinal plants, local taxonomies, and traditional healers play in people’s good health. They also frequently point to the central role of equilibrium—bodily and social—in maintaining people’s well-being. Fewer studies, however, analyze the connection between women’s reproductive health and the broader notions of bodily equilibrium [5,8,9]. This article contributes to this discussion by providing an analysis of two reproductive health issues that reflect Nahua indigenous women’s concerns with bodily equilibrium and its connection to the local sociopolitical context.

A perception of female vulnerability/weakness (*debilidad*) exists in many parts of Mexico. This weakness is seen to be due to the physical make-up of a woman’s body, caused by the loss of menstrual blood, pregnancy, childbirth, and childcare through breastfeeding. Indigenous perceptions see the reproductive life cycle greatly affecting a woman’s body, ultimately diminishing her strength or bodily vigor. Such illnesses include organ displacement and those that waste away a woman’s body.

Organ displacement is an illness category that is found in both present and past cultures of Mexico [5,10,11], the Americas [12,13], and other regions of the world [14,15]. In this illness category, specific organs or parts of the body shift from their original position and cause health problems. The displacement can occur in anyone, from the very young experiencing a fallen fontanelle [16], to women displacing their uterus or pelvic bones [4], to men having displaced testicles, and to both sexes having *me’winik*, a displacement of an abdominal organ identified by the modern Maya [10]. Organ displacement often causes a loss of equilibrium in the person’s body, which must be restored by moving the body part back into its original position.

*Isihuayo* (pronounced: ee-see-HUA-yoh) is the Nahuatl term to describe the “female part.” The term “*sihua*” denotes femaleness in Nahuatl. The *isihuayo* is an organ situated in the lower torso of a woman and is believed to have roots radiating out from it. Though *isihuayo* traditionally described a specific part of the woman’s body [17], over the last few years—especially with the contact that the women have increasingly had with biomedicine—this term is now also equated with two other organs in the lower torso: *matriz* (uterus) and *vejiga* (bladder). (Both these latter terms are in Spanish). This particular illness is known in other parts of Mesoamerica as *caída de matriz*, *baja de matriz*, or *caída de vejiga*[18]. These terms in Spanish describe the actual displacement of the organ.

*Isihuayo* is an ethnomedical category of illness with local cultural and biological characteristics, yet it shares many of the latter characteristics with the biomedical condition of prolapsed uterus [19]. The biomedical symptoms for prolapsed uterus include urinary incontinence, discomfort while urinating, constipation, backache, and a lump that projects outside of the vagina [19]. While prolapsed uterus is a condition identified and treated by biomedicine, the *isihuayo* that the women in the village suffer from differs from prolapse in its etiology and treatment. One of the traditional birth attendants stated that men and women are different because men “do not have ovaries, they have testicles and intestines…[while] we women have the ovaries, the bladder, and the uterus.”

Biomedically, prolapsed uterus is caused when the ligaments holding up the uterus weaken and stretch, allowing it to fall down and hang into the vaginal canal. The etiology is multifactorial, including advanced age, obesity, higher parity (number of births), and conditions causing abdominal pressure [19,20]. Prolapse was diagnosed when the women attended the clinic to have their annual medical exam and Pap smear. The clinicians at the medical centers stated that surgery was typically the treatment for this condition, where the affected parts of the uterus, or even the entire uterus, were removed. In larger hospitals in cities its treatment could include pelvic floor muscle training, hormones, pessaries, or, in severe cases, surgical treatment [19]. The local physicians did not prescribe pessaries, hormones, or exercises, preferring instead to prevent the condition altogether by advising the women to have fewer children and space them further apart.

Another illness that is connected to women’s strength is *necaxantle* (pronounced: neh-cah-SHAN-tleh). *Necaxantle*—“the weakening disease”—is a condition that women develop with improper rest after giving birth [21]. It is locally considered a wasting disease. Ideally, women are expected to rest forty days (*cuarentena*) postpartum so they can regain their bodily strength. Multiple births are believed to increase women’s weakness and decrease her body’s equilibrium, thus making them more susceptible to various illnesses. *Necaxantle* shares many elements with the Andean condition of *sobreparto*. Larme [12] defines *sobreparto* as “confinement illness,” stating that a woman who works in domestic or subsistence activities too soon after giving birth will fall ill. Leatherman [22] adds that because of women’s daily realities, most are unlikely to fully adhere to the postpartum regimen of rest and care. Thus women become especially vulnerable to such conditions.

This article aims to describe the local concepts indigenous Nahua women hold regarding their reproduction. Specifically it addresses the following: (1) Provide a description of two indigenous illnesses—*isihuayo* and *necaxantle*; (2) Discuss the etiology, symptoms, and treatments of both illnesses; and (3) Analyze these illnesses within the local ethnomedical framework and sociopolitical context.

### Study area

Mexico has slightly over 10 million indigenous people in the country, which is approximately 10 percent of the population. The Nahua are the largest indigenous group, numbering nationally around 1.5 million speakers, mostly found in a wide swath across the central Mexican states from the Gulf coast to the Pacific coast. They are the linguistic descendants of the Aztecs. Overall, indigenous populations form part of the lowest socioeconomic status in the country, with the highest levels of ill health, illiteracy, and marginalization [23].

**Figure 1:**
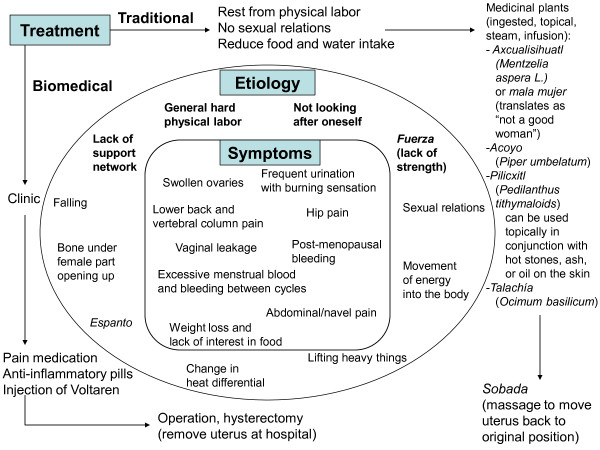
Map of the region of northern Veracruz

This ethnographic and ethnobotanical study was conducted over the course of 13 months (between 2004 and 2007) in a Nahua village in the municipality of Ixhuatlán de Madero, in northern Veracruz, Mexico (Figure 1). With approximately 600 people, the people of the village are primarily subsistence agriculturalists. Their main crop is maize (*Zea mays*), complemented by small-scale cattle ranching and orange-farming.

Northern Veracruz is marked by significant ecological and social contrast. The high Sierra Madre Oriental taper eastwards to rolling hills and a lush coastal zone on the Gulf of Mexico. This region of Ixhuatlán lies on the east side of the mountains and receives abundant rain from the warm, moist winds coming over the Gulf of Mexico that drop their water as they cool while rising over the sierras [24]. The rainfall is usually between 2000 and 2500 millimeters per year [3], with the majo-rity of this rain falling in the form of violent thunderstorms in the wet season from July to November. Only about a third of the precipitation falls during the rest of the year, the dry season. The high rainfall during the wet season has given rise to dense tropical forest in the lower areas and temperate oak-ash forest in the highlands [24,25]. This has led to a high biodiversity of plants and animals. There are also approximately six hundred species of medicinal plants [26]. Some of the plants that are found in this area include avocado (*Persea americana*), ceiba (*Ceiba pentandra*), papaya (*Carica papaya*), datura (*Datura candida*), different types of gourds (*Crescentia alata*), bamboo (*Arthrostylidium racemiflorum*), mahogany (*Swietenia humilis*), guava (*Psidium guajava*), chilies (*Capsicum spp*.), among many others. Many of these plants are domesticated and, though some of them are found in the remaining forest, the majority have been modified by the people and are grown in their home gardens.

Socially, contrast is evident from the marked differences between wealthy landowners and extremely marginalized indigenous populations. Large industrial cities such as Poza Rica are juxtaposed with the rest of the region, which is dotted with small villages and remote hamlets high up in the sierras. Some of the largest cattle ranches in the country, veritable feudal estates [27], are run by a miniscule proportion of the population. The area contains some of the highest rates of illiteracy in the country and a marked lack of services, communications, schools, and jobs. The cities, however, are home to people with a high socioeconomic standard of living [28].

The healthcare in this region consists of biomedicine (government run clinics and hospitals) and traditional healers. The latter include many *sobadores* (bone setters), *curanderos* (ritual specialists), and *parteras* (traditional birth attendants). All the small villages have at least one of these traditional practitioners. In the village where this research takes place, there were nine traditional healers, five of whom were traditional birth attendants (all of them female). All of these women were above the age of 55 and had been practicing most of their adult lives. The *parteras* have received government training and certification in the nearby city of Poza Rica. Through this certification they have been exposed to some of the biomedical terms for the female body.

## Methods

A mixed methodology of participant observation, in-depth ethnographic research (semi-structured and unstructured interviews), and collection of medicinal plants formed the basis of the data. The latter consisted of ex situ interviews that used a combination of fresh material and photographs [29]. The total study population consisted of traditional birth attendants (N = 5), clinicians (N = 8), and laywomen (N = 48). The women ranged in age from 18 to 73, with the average age being 40. The interviews addressed the use of medicinal plants for reproductive purposes, the women’s local understandings of the body, their management of plants in their home gardens, and the enrollment of the women in a large-scale development program.

As noted by Quinlan [30], participant observation is opportunistic, while also allowing the researcher to engage a large proportion of a population in in-depth conversations. In this research, data has been collected from almost 80% of the village’s women and 100% of the traditional birth attendants (TBAs). While interviews co-vered various topics of women’s reproductive health, many of them were self-directed by the participants towards a discussion of illnesses locally known as *isihuayo* and *necaxantle*. *Isihuayo* (uterine displacement/prolapse) affected approximately 20% of the women—though 90% of the sufferers were above the age of 45 and had 5 or more children. *Necaxantle* was much less spoken about. Only 2 informants, both over the age of 55, stated they had suffered from it. The illness was well-known and feared across the village, however.

The qualitative data from the interviews was triangulated and cross-checked. These were sorted into piles and groups, and subsequently analyzed. It was analyzed using open and focused coding by identifying and coding trends and patterns [31].

The collection of the medicinal plants was carried out in conjunction with three of the TBAs and Camila, the project’s local field assistant. Eight of the laywomen mentioned above were informants regarding plant use. All of them were mothers and were between the ages of 35 and 70, with the average age being 51. The fact that they were mothers was important, as it meant that they had experience as the primary caretakers of their family’s health needs. The TBAs were accompanied during their gathering trips to the pastures, agricultural fields, riverbanks, and disturbed areas; plants were collected from people’s home gardens in the village; independent collection was also carried out with the assistance of three key informants. Questions focused particularly on the local uses and taxonomy as well as people’s reasoning behind the use of certain plants. It is important to note that home gardens were women’s domain and thus a large variety of plants frequently grew there, which women could harvest throughout the year and use for purposes such as food, medicine, ornamental, or construction [32].

During this research, the Spanish and Nahuatl name for as many of the plants as possible was obtained; these were cross-checked for accuracy. Many plants had only one name and were not translated into another language. For most plants, the informants provided a local name as well as their taxonomy. The plants collected were scientifically identified by Mtra. Angélica Ramírez Roa at the National Herbarium of Mexico (MEXU) and the voucher specimens were deposited at MEXU and the Herbario del Instituto de Ecología in Xalapa, Veracruz (IE-XAL).

The research was examined and approved by the Institutional Review Boards of the University of Illinois at Chicago and the University of Notre Dame. The research followed internationally recognized ethical guidelines adopted by the American Anthropological Association. Prior informed verbal consent was obtained from all participants before becoming part of the study.

## Results

### Etiology of the illnesses

The etiology of both illnesses is related to a loss of equilibrium in the woman’s body brought about by engaging (or refraining) from certain behavioral norms and expectations.

**Figure 2:**
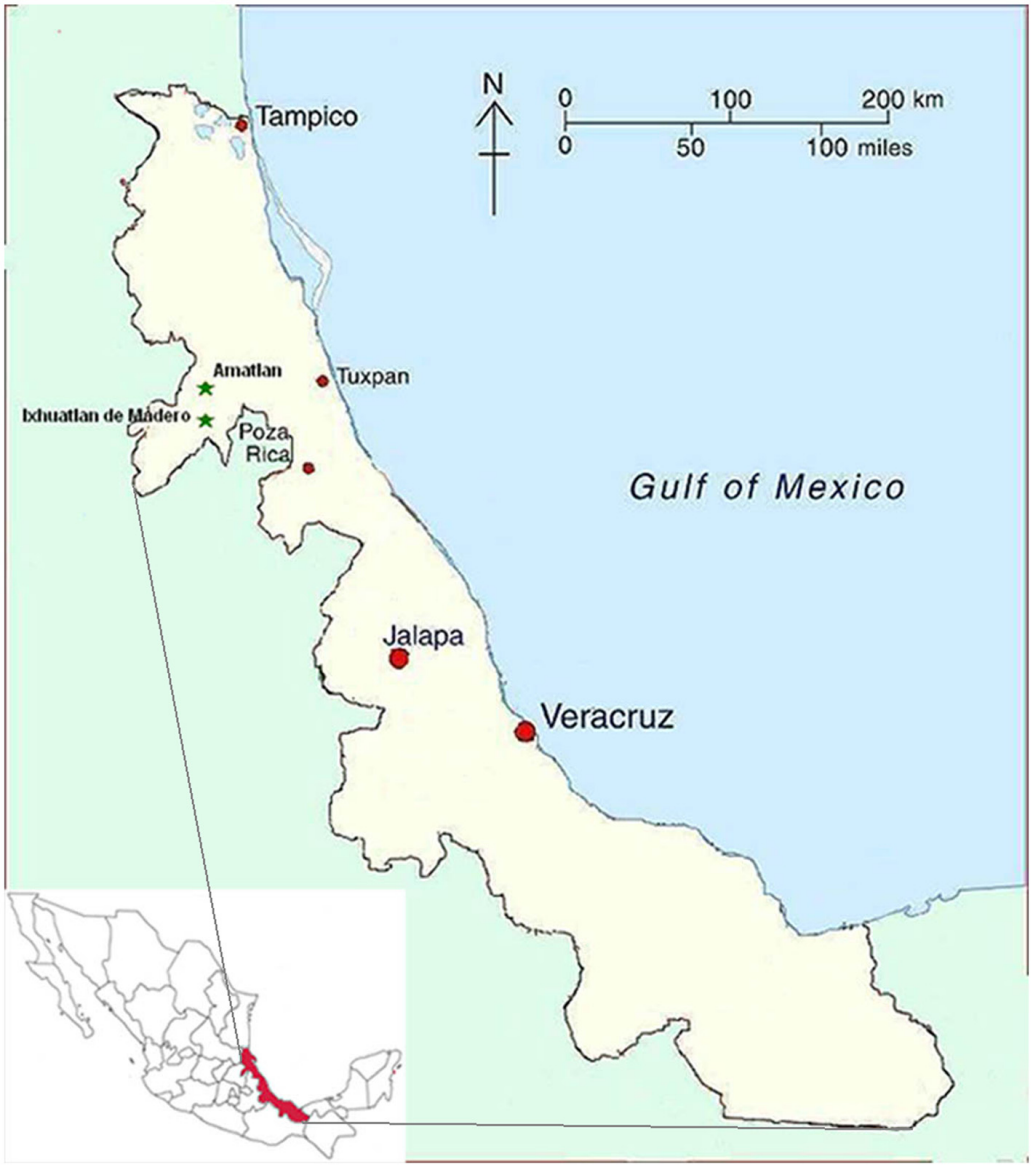
** Etiology, symptoms, and treatment for*isihuayo***

*Isihuayo* can be caused by various ways (Figure 2). If a woman falls or is physically hurt, energy is believed to travel into her body, dislodging her uterus. As one woman, Enriqueta, stated, “It fell a lot when I was pregnant with this [second child]. I could feel it when I moved. I came to Lourdes so she could massage me. That’s how it was.” Carrying heavy loads can affect a woman’s female part over time, this especially the case during pregnancy and immediately postpartum. Refugio, a very sought-after TBA, confirmed the etiology of *isihuayo*. She said about one of her patients, “They say she carries too many buckets and containers of water. And she came to me and said she has pain here [belly] and that is why I massaged her.”

A change in heat differential can also cause *isihuayo*; if, for instance on a hot day a woman is washing clothes in cold water, the internal organs and the body’s heat can drastically change, therefore causing the female part to dislodge. Also, excessive sexual relations, in particularly when the woman is tired, can cause the uterus to loosen from its position and hang down. One of the TBAs stated that if a woman in this situation continues with hard physical labor, the uterus will likely remain in this state.

*Necaxantle* is also caused by imbalance. But this imbalance appears when a postpartum woman does not rest and take care of herself. She does not allow her body to recover. One woman, Esperanza, stated, “[A woman who has just given birth has to rest]. There are some [who rest] for eight days. Others for 15 [days] or a month. But by the next day I was working, as I have no one to help me.” Altagracia confirmed this with her own experience of the illness, “I could no longer look after myself. After four days I began to make tortillas. For my first three children [I rested] a week. Afterwards no longer. I began to work in the kitchen. One can get *necaxantle* [like that].” Among the Mapuche of Chile, for instance, the first few weeks postpartum are considered a period of fragility during which the woman must maintain bodily equilibrium, abstain from sex, and refrain from arduous physical labor [33].

Two important causes of *isihuayo* and *necaxantle* are a woman’s bodily strength (*fuerza*) and her source of social support. Lourdes, one of the TBAs, stated, “You see, women are more delicate than men and so any little bump hurts us. […] It’s just that we all have a small bone [underneath the uterus], and if the legs open [wide] after a fall, it will separate and the bladder falls.” The uterus is believed to fall through the space and hang down, causing it to become tired and begin to hurt. Jordan [4:43] also noted in her research among the Maya the idea of “bones that [open] during childbirth,” which might be a similar etiology to the one experienced by the Nahua women of this region. Lourdes, who, in addition to being a TBA and treating other women’s illnesses, also suffered from *isihuayo*. She said, “I lack *fuerza*; I lack vitamins. With so much work one gets tired. I need medicine, a treatment.” Local perceptions state that a woman’s body becomes weaker as she ages as well as through the difficulties encountered in her reproductive life.

Because of the changing nature of indigenous villages across Mexico, which have experienced out-migration and an increased cash flow, family structures are not completely stable. Most of the women who have suffered *isihuayo* and *necaxantle* have seen their children migrate to cities and border sweatshops, and yet they also become the primary caretakers for their grandchildren. Such a situation leads to a labor-intensive life for these women. They manage all their household domestic duties, oftentimes with little to no help. One of the women stated, “The reason [for *isihuayo*] is not taking care, we have no one to look after us…” This situation places tremendous strain on their bodies, ultimately causing them to weaken and become ill.

All of the women who suffered from *necaxantle* and *isihuayo* expressed their experiences with lack of support. Some of them, such as Juana, were not from the village and so their support came solely from their husband’s family. In Juana’s case, she had seven children but the older ones migrated to work in the cities, leaving her to care for the younger children. For many years she had no support because her mother-in-law worked as a maid in the city and she had no daughters-in-law to help her. Once her eldest son married and his wife came to live in the village, Juana’s health markedly improved. Other women felt the lack of support because they had no daughters to help them. Esperanza’s three daughters lived far from the village, though they left some of their children in her care while they worked as maids or in sweatshops on the U.S.-Mexico border. She attributed her health issues to the hard physical labor—carrying heavy containers of water, for instance—to the lack of support from daughters or daughters-in-law. For others, the hard physical life they led, combined with worry about their family, caused their ill-health. Though Lourdes had a daughter-in-law living next door, she experienced a weakening of her body through a combination of a labor-intensive life as a TBA (and thus constantly traveling to surrounding villages to treat patients) and her constant worry about the welfare of her youngest sons. She felt that *isihuayo* was the underlying cause of her diabetes, which continued to weaken her body, causing even more illness and imbalance.

### Symptoms of these illnesses

Some of the symptoms for *isihuayo* that were mentioned by the women included excessive menstrual blood, bleeding in between menstrual cycles, post-menopausal bleeding, vaginal discharge, weight loss, and swollen ovaries. Abdominal and navel pain are central symptoms. Because the navel is believed to have roots radiating out of it [17:167), its pain radiates to other parts of the body around it. Women mentioned that they knew they had *isihuayo* when they experienced pain in the lower back, lower abdomen, hips, vertebral column, and navel. If a woman had any of these symptoms, she usually suspected that she had the illness, though she would not be certain until she had visited a TBA who would diagnose it after palpating and massaging her abdomen.

Because *necaxantle’s* etiology has such a specific timeframe, if a woman felt any discomfort after birth she would know what ailed her (Table 1). Symptoms include generalized body pain and chills, a feeling of weaknesses, lack of hunger, and weight loss. One woman said, “All her body hurts […] She gets sick, becomes thin. Her body wastes away.” Another concurred, adding, “I felt I had no hunger, and my body started to get very thin. My hands were as [thin] as fingers. My [wrists] were like these fingers.”

**Table 1:** **Etiology, symptoms, and sreatment of*necaxantle***

**Etiology**	**Symptoms**	**Treatment**
Lack of care postpartum	Generalized body pain	Rest from physical labor
Lacking support network	Weight loss	No sexual relations
Lack of *fuerza*	Lack of interest in food	Medicinal plants (bathed)
Bodily imbalance	Chills	
	Weakness	Vitamins

### Treatment for these illnesses

Both *necaxantle* and *isihuayo* are treated with any of the following options: behavioral changes, biomedical compounds, care by a traditional birth attendant, care by biomedical clinicians, and/or use of medicinal plants.

Remedies for *isihuayo* were directed at treating its ultimate cause (the displacement of the organ), rather than solely eliminating the symptoms. As a first step, women were to control the behaviors that caused this condition—abstaining from sexual relations, eating and drinking less (so the uterus was not pressed by the full and heavy bladder, thus being more likely to fall), and reducing physical labor. Figure 2.1 shows some of the active treatments used by the women, particularly those aimed at moving the uterus back to its original position.

Recently-introduced biomedical treatments included pain relief or anti-inflammatory pills and injections to help the uterus settle. Voltaren is a non-steroidal anti-inflammatory drug that works by reducing hormones that cause inflammation and pain in the body. This medical treatment garnered interest in recent years as the TBAs co-opted it for themselves without necessarily knowing what the medication was originally intended for. Altagracia, one of the women, commented,

“[Juana] said that she cured herself. That just by taking a plant she got better. [She said] she would drink it like water all day and that she got better. But that plant is far away and none grow near here. It is difficult to go for it. That woman’s husband helped her [to get it]. If you look for that plant it sticks to your clothes and you can’t get it off. That’s probably why it works. She said that just with that she got better, that the [uterus] lifted back [into place]. But the doctor says that nothing will cure it, that neither massages not plants work. [He says] only surgery works.”

Regardless of whether the woman used plants or allopathic medication, the uterus had to be manually moved back to its original position by a traditional birth attendant. The TBA would carry out a *sobada* (traditional massage) on the woman’s abdomen and slowly settle the displaced organ back into its place.

Women aimed to prevent *necaxantle* altogether, though they agreed that this was not always easy to manage because of the increased domestic duties women had. Rest was the immediately prescribed solution. Additionally, a strong belief in the power of vitamins exists throughout Mexico [34]. Vitamin-laden liquids are sold in all pharmacies in the countries and are used for any illness one might have. They are seen to counteract bodily weakness and return strength to the person. Esperanza stated, “They gave me vitamins, only vitamins was I given. And like that it went away.”

### Medicinal plants used

Medicinal plants are a central part of the treatment for *isihuayo* and *necaxantle* (Table 2). This research project collected 170 plants used in this Nahua community, 80 of which were used medicinally. A total of 26 plants were used to treat issues related to reproduction. Of these plants, 4 were used for *isihuayo*, while only 1 was used for *necaxantle*. While most people had a basic knowledge about medicinal plants, the people with the greatest knowledge were women (especially those who were married and had children) and traditional healing specialists.

**Table 2:** **Medicinal plants used for treating isihuayo and*necaxantle***

**Local name**	**Scientific name**	**Family**	**Uses**
Mala mujer	*Mentzelia aspera* L.	Loasaceae	Tea
Talachía	*Ocimum basilicum* L.	Lamiateae	Steam
Pilicxitl	*Pedilanthus tithymaloides* (L.) Poit.	Euphorbiaceae	Topical
Acoyo	*Piper umbellatum* L.	Piperaceae	Steam/Topical
Necaxancuamekatl	*Solanum wendlandii* Hook f.	Solanaceae	Bath

Plants that are “sticky” or help the uterus “adhere” to its correct position were vital for *isihuayo*. One such plant is *Mentzelia aspera* L., which was used in the form of an infusion/tea. This plant was known locally as *axcualisihuatl* (in Nahuatl) or *mala mujer* (in Spanish); this translates as “bad woman.” The leaves of this plant adhere to skin and clothing with ease; it is this property that was considered to help the uterus adhere to where it belongs.

Another treatment was to squat over a steaming infusion of either *acoyo* (*Piper umbellatum* L.) or *talachía* (*Ocimum basilicum* L.) so the steam could help a woman’s uterus dry up and to settle back into place [21]. Bussman and Glenn [35] note the use of *Ocimum basilicum* in Peru for reproductive purposes, primarily related to uterine health postpartum.

Refugio, a TBA, stated, “[A woman should put] hot water in a container and […] steam her parts (raises skirt and pretends to squat over a pretend container), there where the uterus comes out. Because […] lots of water [comes] out, and […] it’s because the uterus wants to come out. […] This [treatment] is so that the uterus dries up.” Some plants, *acoyo* (*Piper umbellatum* L.), or *pilicxitl* (*Pedilanthus tithymaloides* (L.) Poit.) were also used topically in conjunction with hot stones, ash, or oil on the skin above the root of the uterus to settle it back into place. Refugio confirmed that these plants helped the uterus to become stronger and harder so it could return to its original position and be able to withstand other problems in the future. The equilibrium in the woman’s body was restored through these treatments [21]. Giovannini and Heinrich [7], in their work on medicinal plants among the Mazatec of Mexico, note the use of *Piper umbellatum*. In this group this plant is used for body pains and nausea rather than for uterine health. They also note the use of *Pedilanthus tithymaloides* for sore throat and cough.

Only one medicinal plant was used for *necaxantle*. It is locally called *necaxancuamekatl* (*Solanum wendlandii* Hoof. f.)—meaning “plant for *necaxantle*.” A woman who suffered from this illness would bathe with this plant; she would prepare it by crushing it and mixing it with well water. She would bathe with this several times a day until she felt that her symptoms of weakness and lack of hunger disappeared. The woman would accompany this treatment with rest so her body could return to normal. Traditional birth attendants also were sought out for this illness. *Necaxancuamekatl* was not easy to obtain, growing only rarely and in inaccessible places. If a woman needed such a treatment, her relatives would often walk for many miles to another village searching for the plant.

## Discussion

Across Mesoamerica exists the humoral system, consisting of a binary opposition between hot-cold and dry-wet [36]. This constitution does not refer to the actual temperature of a substance but rather refers to characteristics of their temporal nature [37,38]. A healthy body is one that has warmth evenly distributed throughout; imbalances may occur, however, due to illness and disturbance of a person's equilibrium by supernatural or natural forces. An imbalance may be of a hot or a cold nature [36] and the medicinal plants chosen to cure the imbalance will be based on the "use of opposites" [1:14]. Hence, a cold plant will be used to cure a hot illness, while a hot one will be used for a cold illness. Many of the treatments for *isihuayo* or *necaxantle* used by the Nahua women of this study seem to have a hot temporal quality. The plants are temporally hot and they are also used in conjunction with heating treatments, such as the steam and hot stones. This use is because both *isihuayo* and *necaxantle*, by their weakening nature, are cold illnesses. The increase in both humoral and actual temperatures therefore help the women’s reproductive body return to equilibrium.

Childbirth complications continue to exact a major toll on women’s health worldwide. Most small-scale societies around the world carry out practices to protect women’s reproductive health. Among the Kry of Laos, for instance, several steps are taken to protect the woman and newborn infant, such as diets, baths, and use of medicinal plants [39]. The Mapuche of Chile have various postpartum behavioral restrictions for the woman to regain equilibrium [33]. In many of these societies, the role of traditional birth attendants is central to women’s health, such as in Guatemala [18], Belize [40], Mozambique [41], or Malaysia [42], to name a few.

Leatherman [22:61], in his analysis of reproductive illnesses in the Andes, proposes an interesting reason for women’s poor reproductive health. He states that reproductive illnesses become an accepted reason for reducing one’s domestic workload or sexual relations with spouses—they thus allow women to “negotiate some limited control over their productive and reproductive roles.” With this view, then, one could conceivable see *necaxantle* or *isihuayo* in a different light, perhaps as ways for women to exact some support from relatives or friends in times of need.

In epidemiological terms, these illnesses fall under reproductive morbidity, both obstetric (postpartum issues with *necaxantle*) and gynecological (prolapsed uterus). Due to issues with cross-cultural definition, underreporting, or the relationship of the women to the larger medical institutions of the nation state there is no worldwide understanding of the way that women from small-scale societies experience these illnesses [43].

## Conclusions

Uterine health concerns occupy a significant portion of the conversations of the Nahua women of this study. Though only a portion had suffered from *isihuayo* or *necaxantle*, many women feared these illnesses and spoke about their efforts at prevention. Many of these fears were centered upon the women’s workload and feelings of lack of support, which had only increased over the past few decades as the village’s structure changed. Over the past few decades, the number of people (especially young women) migrating to the cities has rapidly increased. This situation not only changes the demographics of the village, but also contributes to a shift in older women’s responsibilities regarding the domestic sphere.

Though most rural-living Nahua women worked very hard cooking, washing clothes, collecting firewood and water, or tending farm animals, if they lacked additional hands to share the load (in the form of children, siblings, or parents) their work load became heavier and they were more likely to suffer from a bodily imbalance due to exhaustion or weakness. In the case of *isihuayo*, a woman perhaps had no grown sons to carry heavy loads of water or firewood and no daughters to help with cooking and washing clothes. Thus the overwork led to her weakness and, if one day she happened to fall down, her uterus would likely become dislodged and displaced. The same was true for *necaxantle*, where if a woman gave birth but had no social support in the form of other women in the household to take on her chores, she had no choice but to begin working soon afterward. Her body thus had no time to recover and so became imbalanced.

The research on these two ethnomedical conditions is a useful case study to understanding how indigenous women experience reproductive health. Reproductive health is not simply about clinically-based medicine (fertility control, prenatal care, or birth options) but is also how this medical system intersects with the local bodily concepts. By describing and analyzing indigenous women’s ill health, one can focus upon the combination of causes—which extend beyond the physical body and into the larger structure that the women exist in. Thus, social, economic, and political factors all shape the women’s experiences of poor reproductive health. They are indigenous women, and thus at the bottom strata of Mexican social/economic hierarchy. They face a changing social structure, brought about by migration, economic welfare programs, and changing medical landscape [9]. And ultimately, their domestic duties and workloads take a toll on their bodies, making them susceptible to ill health.

## Author’s contributions

All research herein was designed, conducted, and authored by the author.

## References

[B1] Browner CH Criteria for selecting herbal remedies Ethnology 1985 24 13 32 10.2307/3773487

[B2] Farfán Morales O Suárez y Farías MC Los nahuas de la sierra norte de Puebla: el chamanismo entre los nahuas Estudios nahuas 1988 INAH, México 127 144

[B3] Sandstrom AR Corn is our Blood: Culture and Ethnic Identity in a Contemporary Aztec Village 1991 University of Oklahoma Press, Norman

[B4] Jordan B Birth in Four Cultures: A Crosscultural Investigation of Childbirth in Yucatán, Holland, Sweden, and the United States 1993 Waveland Press, Inc., Prospect Heights, IL

[B5] Castañeda-Camey X García-Barrios C Romero-Guerrero X Nuñez-Urquiza RM Gonzalez-Hernández D Glass AL Traditional birth attendants in Mexico: advantages and inadequacies of care for normal deliveries Soc Sci Med 1996 43 199 207 10.1016/0277-9536(95)00362-2 8844924

[B6] Weimann C Heinrich M Indigenous medicinal plants in Mexico: The example of the Nahua (Sierra de Zongolica) Botanica Acta 1997 110 62 72

[B7] Giovannini P Heinrich M Xki yoma’ (our medicine) and xki tienda (patent medicine)—interface between traditional and modern medicine among the Mazatecs of Oaxaca, Mexico J Ethnopharmacol 2009 121 383 399 10.1016/j.jep.2008.11.003 19041707

[B8] Groark KP Vital warmth and well-being: steam bathing as household therapy among the Tzeltal and Tzotzil Maya of highland Chiapas, Mexico Soc Sci Med 2005 61 785 795 10.1016/j.socscimed.2004.08.044 15950091

[B9] Smith-Oka V Unintended consequences: exploring the tensions between development programs and indigenous women in Mexico in the context of reproductive health Soc Sci Med 2009 68 2069 2077 10.1016/j.socscimed.2009.03.026 19362404

[B10] Berlin EA Jara VM Berlin B Breedlove DE Duncan TO Laughlin RM Me'winik: discovery of the biomedical equivalence for a Maya ethnomedical syndrome Soc Sci Med 1993 37 671 678 10.1016/0277-9536(93)90106-E 8211281

[B11] Fuller N Jordan B Maya women and the end of the birthing period: postpartum massage-and-binding in Yucatán, Mexico Med Anth 1981 5 35 50 10.1080/01459740.1981.9986974 22273103

[B12] Larme AC Environment, vulnerability, and gender in Andean ethnomedicine Soc Sci Med 1998 47 1005 1015 10.1016/S0277-9536(98)00162-2 9723847

[B13] Singer M Davison L Gerdes G Culture, critical theory, and reproductive illness behavior in Haiti Med Anthropol Q 1988 2 370 385 10.1525/maq.1988.2.4.02a00060

[B14] Meyer CL The Wandering Uterus: Politics of Reproductive Rights of Women 1997 New York University Press, New York

[B15] Earth B Sthapit S Uterine prolapse in rural Nepal: gender and human rights implications Culture, Health & Sexuality 2002 4 281 296 10.1080/13691050110090248

[B16] Hinojosa SZ The Mexican American sobador, convergent disease discourse, and pain validation in south Texas Hum Org 2008 67 194 206

[B17] López Austin A Ortiz de Montellano T, Ortiz de Montellano B The Human Body and Ideology Concepts of the Ancient Nahuas, Vol. I 1988 University of Utah Press, Salt Lake City

[B18] Cosminsky S Huber BR, Sandstrom AR Maya midwives in southern Mexico and Guatemala Mesoamerican Healers 2001 University of Texas Press, Austin 179 210

[B19] Doshani A Teo REC Mayne CJ Tincello DG Uterine prolapse B Med J 2007 335 819 823 10.1136/bmj.39356.604074.BE PMC2034734 17947787

[B20] Mant J Painter R Vessey M Epidemiology of genital prolapse: observations from the Oxford family planning association study B J Obs Gyn 1997 104 579 585 10.1111/j.1471-0528.1997.tb11536.x 9166201

[B21] Smith-Oka V Plants used for reproductive health by Nahua women in northern Veracruz, Mexico J Econ Bot 2008 62 604 614 10.1007/s12231-008-9026-7

[B22] Leatherman T A space of vulnerability in poverty and health: political-ecology and biocultural analysis Ethos 2005 33 46 70 10.1525/eth.2005.33.1.046

[B23] Comisión Nacional Para el Desarrollo de los Pueblos Indígenas Indicadores y estadísticas CDI, Mexico City http://www.cdi.gob.mx/index.php?option=com_content&view=category&id=38&Itemid=54

[B24] Escobar Ohmstede A Historia de los Pueblos Indígenas de México: De la Costa a la Sierra, Las Huastecas 1998 CIESAS-INI, México 1750 1900

[B25] Sandstrom AR The image of disease: medical practices of Nahua Indians of the Huasteca University of Missouri Monographs in Anthropology, No. 3. Department of Anthropology 1978 University of Missouri-Columbia, Columbia

[B26] Avendaño Reyes S Castillo-Campos G, Mejía-Saulés MT El conocimiento de la flora útil: una base para conservar los recursos vegetales Problemática Ambiental en el Estado de Veracruz: Los Recursos Vegetales 1994 Universidad Veracruzana, Xalapa, México 59 67

[B27] Ariel de Vidas A La bella durmiente: el norte de Veracruz 2005 Mundos Nuevos, Nuevo Mundo http://nuevomundo.revues.org/574

[B28] Ruvalcaba Mercado J Ruvalcaba Mercado J Presentación Nuevos aportes al conocimiento de la Huasteca 1998 CIESAS, México 11 26

[B29] Thomas E Vandebroek I Van Damme P What works in the field? a comparison of different interviewing methods in ethnobotany with special reference to the use of photographs Econ Bot 2007 614 376 384

[B30] Quinlan MB Ethnomedicine and ethnobotany of fright, a Caribbean culture-bound psychiatric syndrome J Ethnobiol Ethnomed 2010 6 9 10.1186/1746-4269-6-9 20163730 PMC3583188

[B31] Emerson RM Fretz RI Shaw LL Writing Ethnographic Fieldnotes 1995 University of Chicago Press, Chicago

[B32] Vázquez-García V Gender, ethnicity, and economic status in plant management: un- cultivated edible plants among the Nahua and Popolucas of Veracruz, Mexico Agri Hum Values 2008 25 65 77

[B33] Alarcón AM Nahuelcheo Y Pregnancy, delivery, and post partum beliefs among Mapuche women: private conversations Chungara, Revista de Antropologiá Chilena 2008 40 193 202

[B34] Finkler K Biomedicine globalized and localized: western medical practices in an outpatient clinic of a Mexican hospital Soc Sci Med 2004 59 2037 2051 10.1016/j.socscimed.2004.03.008 15351471

[B35] Bussmann RW Glenn A Medicinal plants used in Northern Peru for reproductive problems and female health J Ethnobiol Ethnomed 2010 6 30 10.1186/1746-4269-6-30 21040536 PMC2984435

[B36] Rubel AJ Hass MR Sargent CF, Johnson TM Ethnomedicine Medical anthropology: contemporary theory and method. Revised edition 1996 Praeger Publishers, Westport 113 130

[B37] López Austin A Viesca Treviño C Cosmovisión y medicina náhuatl Estudios sobre botánica y antropología médica 1976 Instituto Mexicano para el Estudio de las Plantas Medicinales, Mexico 13 27

[B38] Etkin NL Sargent CF, Johnson TM Ethnopharmacology: the conjunction of medical ethnography and the biology of therapeutic action Medical anthropology: contemporary theory and method. Revised edition 1996 Praeger Publishers, Westport 151 164

[B39] Lamxai V de Boer HJ Björk L Traditions and plant use during pregnancy, childbirth and postpartum recovery by the Kry ethnic group in Lao PDR J Ethnobiol Ethnomed 2011 7 14 10.1186/1746-4269-7-14 21569234 PMC3120637

[B40] Maraesa A Fordyce L, Maraesa A A competition over reproductive authority: prenatal risk assessment in southern Belize Risk, reproduction, and narratives of experience 2012 Vanderbilt University Press, Nashville

[B41] Chapman R Chikotsa—secrets, silence, and hiding: social risk and reproductive vulnerability in central Mozambique Med Anth Quar 2006 20 487 515 10.1525/maq.2006.20.4.487 17225656

[B42] Manderson L Ram K, Jolly M Shaping reproduction: maternity in early twentieth century Malaya Maternities and modernities: colonial and postcolonial experiences in Asia and the Pacific 1998 Cambridge University Press, Cambridge 26 50

[B43] Blanchard K Elul B RamaRao S Reproductive Health Indicators: Moving Forward 1999 Population Council, New York

